# Supplementary material for: Resolving the full spectrum of human genome variation using Linked-Reads
Source: Genome Res. 2019 Apr;29(4):635–45. doi: 10.1101/gr.234443.118 (PMC6442396; doi:10.1101/gr.234443.118)
Supplement: Supplemental Material [file supp_gr.234443.118_Supplemental_longranger-2.2.zip › longranger-2.2.tar.bz2!/lr2.2-public/lib/stan/cmdstan/stan/doc/api/html/agrad_2rev_2matrix_2squared__distance_8hpp_source.html]

Stan: src/stan/agrad/rev/matrix/squared\_distance.hpp Source File


|  |  |
| --- | --- |
|  | Stan  2.5.0  probability, sampling & optimization |


- Main Page
- Related Pages
- Namespaces
- Classes
- Files

- File List
- File Members

All Classes Namespaces Files Functions Variables Typedefs Enumerations Enumerator Friends Macros Pages

- src
- stan
- agrad
- rev
- matrix

squared\_distance.hpp

Go to the documentation of this file.

1 #ifndef STAN\_\_AGRAD\_\_REV\_\_MATRIX\_\_SQUARED\_DISTANCE\_HPP

2 #define STAN\_\_AGRAD\_\_REV\_\_MATRIX\_\_SQUARED\_DISTANCE\_HPP

3

4 #include <vector>

5

6 #include <stan/agrad/rev/var.hpp>

7 #include <stan/agrad/rev/vari.hpp>

8 #include <stan/agrad/rev/functions/sqrt.hpp>

9 #include <stan/agrad/rev/matrix/typedefs.hpp>

10

11 #include <stan/math/error\_handling/matrix/check\_vector.hpp>

12 #include <stan/math/error\_handling/matrix/check\_matching\_sizes.hpp>

13 #include <stan/math/matrix/Eigen.hpp>

14 #include <stan/math/matrix/typedefs.hpp>

15 #include <stan/math/matrix/meta/index\_type.hpp>

16 #include <stan/math/meta/index\_type.hpp>

17

18

19 namespace stan {

20

21  namespace agrad {

22

23  namespace {

24

25  class squared\_distance\_vv\_vari : public vari {

26  protected:

27  vari\*\* v1\_;

28  vari\*\* v2\_;

29  size\_t length\_;

30

31  template <int R1,int C1,int R2,int C2>

32  inline static double

33  var\_squared\_distance(const Eigen::Matrix<var,R1,C1> &v1,

34  const Eigen::Matrix<var,R2,C2> &v2) {

35  using Eigen::Matrix;

36  using stan::math::index\_type;

37  typedef typename index\_type<Matrix<var,R1,R2> >::type idx\_t;

38  double result = 0;

39  for (idx\_t i = 0; i < v1.size(); i++) {

40  double diff = v1(i).vi\_->val\_ - v2(i).vi\_->val\_;

41  result += diff\*diff;

42  }

43  return result;

44  }

45  public:

46  template<int R1,int C1,int R2,int C2>

47  squared\_distance\_vv\_vari(const Eigen::Matrix<var,R1,C1> &v1,

48  const Eigen::Matrix<var,R2,C2> &v2)

49  : vari(var\_squared\_distance(v1, v2)), length\_(v1.size())

50  {

51  v1\_ = (vari\*\*)memalloc\_.alloc(length\_\*sizeof(vari\*));

52  for (size\_t i = 0; i < length\_; i++)

53  v1\_[i] = v1(i).vi\_;

54

55  v2\_ = (vari\*\*)memalloc\_.alloc(length\_\*sizeof(vari\*));

56  for (size\_t i = 0; i < length\_; i++)

57  v2\_[i] = v2(i).vi\_;

58  }

59  virtual void chain() {

60  for (size\_t i = 0; i < length\_; i++) {

61  double di = 2 \* adj\_ \* (v1\_[i]->val\_ - v2\_[i]->val\_);

62  v1\_[i]->adj\_ += di;

63  v2\_[i]->adj\_ -= di;

64  }

65  }

66  };

67  class squared\_distance\_vd\_vari : public vari {

68  protected:

69  vari\*\* v1\_;

70  double\* v2\_;

71  size\_t length\_;

72

73  template<int R1,int C1,int R2,int C2>

74  inline static double

75  var\_squared\_distance(const Eigen::Matrix<var,R1,C1> &v1,

76  const Eigen::Matrix<double,R2,C2> &v2) {

77

78  using Eigen::Matrix;

79  using stan::math::index\_type;

80  typedef typename index\_type<Matrix<double,R1,C1> >::type idx\_t;

81

82  double result = 0;

83  for (idx\_t i = 0; i < v1.size(); i++) {

84  double diff = v1(i).vi\_->val\_ - v2(i);

85  result += diff\*diff;

86  }

87  return result;

88  }

89  public:

90  template<int R1,int C1,int R2,int C2>

91  squared\_distance\_vd\_vari(const Eigen::Matrix<var,R1,C1> &v1,

92  const Eigen::Matrix<double,R2,C2> &v2)

93  : vari(var\_squared\_distance(v1, v2)), length\_(v1.size())

94  {

95  v1\_ = (vari\*\*)memalloc\_.alloc(length\_\*sizeof(vari\*));

96  for (size\_t i = 0; i < length\_; i++)

97  v1\_[i] = v1(i).vi\_;

98

99  v2\_ = (double\*)memalloc\_.alloc(length\_\*sizeof(double));

100  for (size\_t i = 0; i < length\_; i++)

101  v2\_[i] = v2(i);

102  }

103  virtual void chain() {

104  for (size\_t i = 0; i < length\_; i++) {

105  v1\_[i]->adj\_ += 2 \* adj\_ \* (v1\_[i]->val\_ - v2\_[i]);

106  }

107  }

108  };

109  }

110

111  template<int R1,int C1,int R2, int C2>

112  inline var squared\_distance(const Eigen::Matrix<var, R1, C1>& v1,

113  const Eigen::Matrix<var, R2, C2>& v2) {

114  stan::math::check\_vector("squared\_distance(%1%)",v1,"v1",(double\*)0);

115  stan::math::check\_vector("squared\_distance(%1%)",v2,"v2",(double\*)0);

116  stan::math::check\_matching\_sizes("squared\_distance(%1%)",v1,"v1",

117  v2,"v2",(double\*)0);

118  return var(new squared\_distance\_vv\_vari(v1,v2));

119  }

120  template<int R1,int C1,int R2, int C2>

121  inline var squared\_distance(const Eigen::Matrix<var, R1, C1>& v1,

122  const Eigen::Matrix<double, R2, C2>& v2) {

123  stan::math::check\_vector("squared\_distance(%1%)",v1,"v1",(double\*)0);

124  stan::math::check\_vector("squared\_distance(%1%)",v2,"v2",(double\*)0);

125  stan::math::check\_matching\_sizes("squared\_distance(%1%)",v1,"v1",

126  v2,"v2",(double\*)0);

127  return var(new squared\_distance\_vd\_vari(v1,v2));

128  }

129  template<int R1,int C1,int R2, int C2>

130  inline var squared\_distance(const Eigen::Matrix<double, R1, C1>& v1,

131  const Eigen::Matrix<var, R2, C2>& v2) {

132  stan::math::check\_vector("squared\_distance(%1%)",v1,"v1",(double\*)0);

133  stan::math::check\_vector("squared\_distance(%1%)",v2,"v2",(double\*)0);

134  stan::math::check\_matching\_sizes("squared\_distance(%1%)",v1,"v1",

135  v2,"v2",(double\*)0);

136  return var(new squared\_distance\_vd\_vari(v2,v1));

137  }

138  }

139 }

140 #endif

vari.hpp

typedefs.hpp

typedefs.hpp

stan::agrad::memalloc\_

memory::stack\_alloc memalloc\_

**Definition:** var\_stack.cpp:16

stan::math::check\_vector

bool check\_vector(const char \*function, const Eigen::Matrix< T, R, C > &x, const char \*name, T\_result \*result)

**Definition:** check\_vector.hpp:14

check\_matching\_sizes.hpp

stan::math::index\_type

Primary template class for the metaprogram to compute the index type of a container.

**Definition:** index\_type.hpp:21

v2\_

vari \*\* v2\_

**Definition:** squared\_distance.hpp:28

length\_

size\_t length\_

**Definition:** squared\_distance.hpp:29

index\_type.hpp

var.hpp

sqrt.hpp

Eigen.hpp

stan::memory::stack\_alloc::alloc

void \* alloc(size\_t len)

Return a newly allocated block of memory of the appropriate size managed by the stack allocator...

**Definition:** stack\_alloc.hpp:168

check\_vector.hpp

stan::agrad::var

Independent (input) and dependent (output) variables for gradients.

**Definition:** var.hpp:27

stan::math::size

int size(const std::vector< T > &x)

**Definition:** size.hpp:11

stan::agrad::squared\_distance

var squared\_distance(const Eigen::Matrix< var, R1, C1 > &v1, const Eigen::Matrix< var, R2, C2 > &v2)

**Definition:** squared\_distance.hpp:112

stan::math::check\_matching\_sizes

bool check\_matching\_sizes(const char \*function, const T\_y1 &y1, const char \*name1, const T\_y2 &y2, const char \*name2, T\_result \*result)

**Definition:** check\_matching\_sizes.hpp:15

index\_type.hpp

v1\_

vari \*\* v1\_

**Definition:** squared\_distance.hpp:27


---

[ Stan Home Page ]

*© 2011–2014,
Stan Development Team.*
